# Case Report: A case of severe mosquito bite allergy in an elderly patient

**DOI:** 10.3389/fimmu.2026.1789094

**Published:** 2026-04-22

**Authors:** Huizhong Wang, Jia Chen, Jingwen Yang, Yun Xia, Liuqing Chen, Bin Hu

**Affiliations:** 1 Department of Dermatology, Wuhan No. 1 Hospital, Wuhan, China; 2 Hubei Province & Key Laboratory of Skin Infection and Immunity, Wuhan No. 1 Hospital, Wuhan, China; 3 Department of Dermatology, Traditional Chinese and Western Medicine Hospital of Wuhan, Tongji Medical College, Huazhong University of Science and Technology, Wuhan, China

**Keywords:** chronic active Epstein-Barr virus disease (CAEBVD), Epstein-Barr virus (EBV), hypersensitivity to mosquito bites (HMB), lymphoproliferative disorders (LPD), severe mosquito bite allergy (SMBA)

## Abstract

**Background:**

Severe mosquito bite allergy (SMBA) is characterized by intense local necrotic skin reactions accompanied by systemic symptoms following mosquito bites. SMBA is currently classified within the spectrum of EBV-positive T/natural killer (NK)-cell lymphoproliferative disorders (LPDs) and is recognized as a specific cutaneous manifestation of chronic active EBV disease (CAEBVD), with a potential risk of progression to overt T/NK-cell leukemia or lymphoma.

**Case presentation:**

Herein, we report a rare case of a 70-year-old male diagnosed with EBV-associated NK-cell LPD manifesting primarily as SMBA. The patient presented with recurrent disseminated erythematous plaques, bullae, necrotic ulcers, and scarring following mosquito bites, accompanied by intermittent fever. Quantitative PCR revealed a high EBV DNA load in peripheral blood, and skin biopsy demonstrated an angiocentric infiltration of EBV-positive NK cells. Based on the clinical features of SMBA, high EBV load, and histopathological findings, the diagnosis of EBV-associated NK-cell LPD was established. Treatment with oral prednisone and methotrexate resulted in significant clinical improvement, and the patient maintained a stable, indolent course during the subsequent 2-year follow-up.

**Conclusion:**

We report a rare case of elderly-onset EBV-associated NK-cell LPD manifesting as SMBA, characterized by an unusually indolent clinical course. Furthermore, we provide a comprehensive literature review of 9 previously reported adult SMBA cases to offer insights for clinical practice.

## Introduction

EBV is a ubiquitous human herpesvirus that infects over 95% of the adult population worldwide (1). CAEBVD is a systemic lymphoproliferative disorder characterized by the clonal proliferation of EBV-infected T or NK cells and their infiltration into various organs, resulting in persistent inflammatory symptoms and multiple organ damage. SMBA is a rare cutaneous disease, considered a cutaneous manifestation of EBV-associated NK cell LPDs and closely related to CAEBVD (2–5).

Clinically, SMBA is defined by intense local skin reactions precipitated by mosquito bites, accompanied by systemic constitutional symptoms such as high fever and lymphadenopathy (2). The condition carries a significant risk of progression to life-threatening systemic diseases, including hemophagocytic lymphohistiocytosis (HLH) and overt EBV-associated malignancies (6–8). While SMBA predominantly affects children and adolescents, adult-onset cases are exceedingly rare (9). Here, we report a rare case of an elderly male diagnosed with EBV-associated NK-cell LPD, which is characterized by SMBA as the primary clinical presentation and an unusually indolent course. This case report was written in accordance with the CARE (Case Reports) guidelines (https://www.care-statement.org/checklist).

## Case presentation

**Figure 1:**
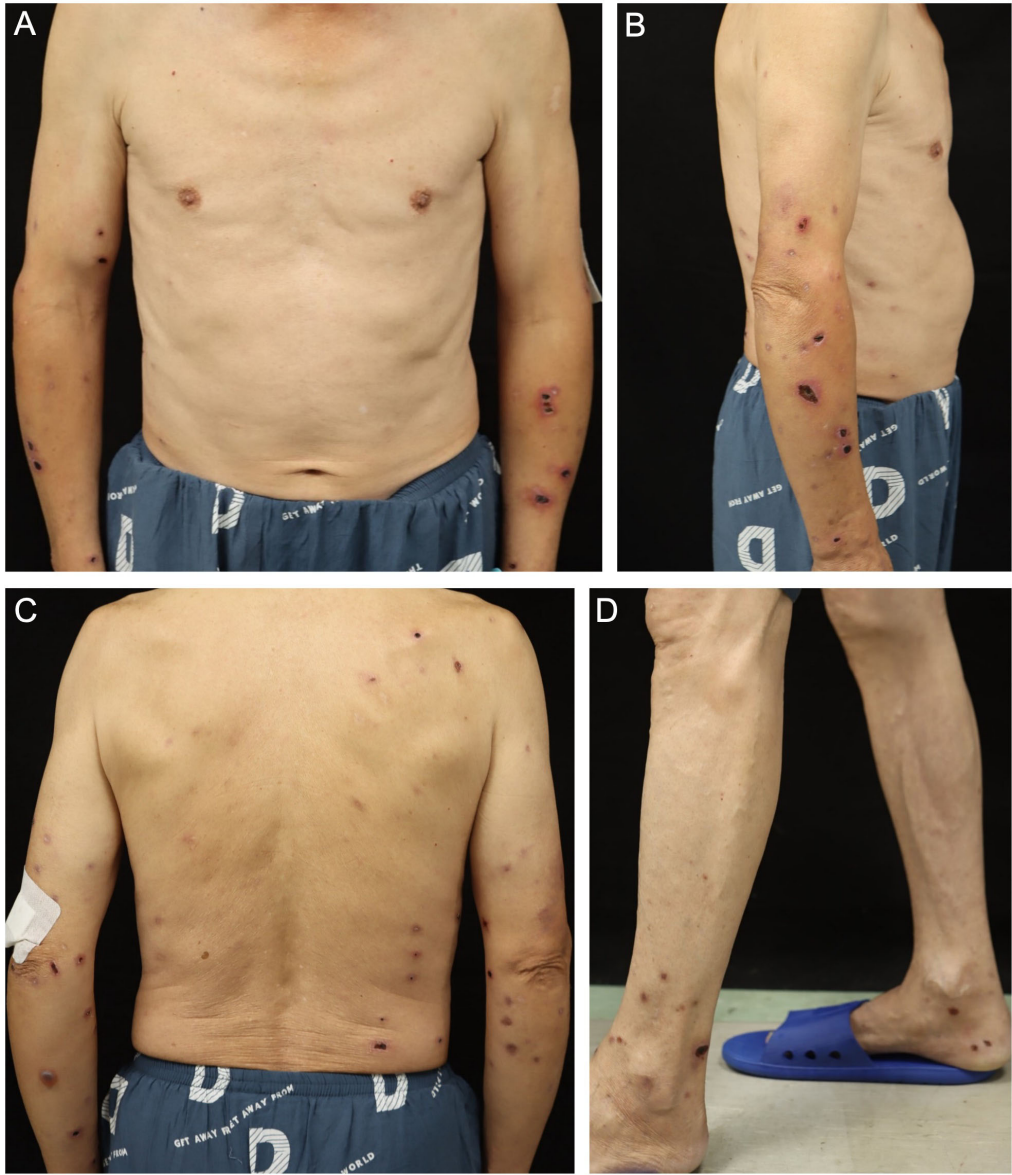
Cutaneous presentation of the patient. **(A, B)** Ulcerative, tender lesions surrounded by soft tissue swelling on the upper extremities. **(C)** Blood-filled bullae on the left upper arm and depressed scars on the back and upper extremities. **(D)** Depressed scars and ulcerative lesions dried out with scabbing on the lower extremities.

A 70-year-old male presented to our clinic with a 3-year history of recurrent scattered erythema, bullae, ulcers, and scarring following mosquito bites in the summer. Two months prior to presentation, the skin lesions were markedly exacerbated following mosquito bites, accompanied by pain, intense pruritus and intermittent fever. The highest recorded body temperature was 37.8 °C, accompanied by chills and headache. He had no significant past medical or family history. Physical examination revealed numerous areas of gangrenous erythema, blood-filled bullae, depressed scars and ulcerative lesions surrounded by soft tissue swelling on the back and distal extremities. Some of the skin lesions exhibited central necrosis with hemorrhagic and black eschars (**Figure 1**).

Complete blood count revealed a total white blood cell (WBC) count of 8.46 × 10^9/L (normal range, 3.5-9.5 × 10^9/L), accompanied by lymphocytosis (6.83 × 10^9/L; normal range, 1.1-3.2 × 10^9/L), neutropenia (1.1 × 10^9/L; normal range, 1.8-6.3 × 10^9/L), and thrombocytopenia (72 × 10^9/L; normal range, 100-300 × 10^9/L), with a hemoglobin level of 105 g/L (normal range, 130–175 g/L). Peripheral blood flow cytometry further phenotyped the lymphocytosis, demonstrating a normal T-cell count of 967.76 × 10^6/L (normal range, 723-2271 × 10^6/L) and a profoundly elevated NK-cell count (5223.32 × 10^6/L; normal range, 61-607 × 10^6/L). Additional laboratory assessments showed hyperferritinemia (1427.0 ng/mL; normal range, 24–425 ng/mL), elevated IgE (215.0 IU/mL; normal range, 0.1–200 IU/mL), and hypofibrinogenemia (1.36 g/L; normal range, 2–4 g/L), while triglyceride levels were within the normal reference range (1.08 mmol/L; normal range, 0-1.7 mmol/L). Quantification of Epstein-Barr virus (EBV) DNA showed high loads of virus in the peripheral blood (8483 copies/mL; normal range, <400 copies/mL). Ultrasonography detected slightly lymphadenopathies on bilateral cervical, axillary, and inguinal lymph nodes, with the largest lymph node measuring approximately 2.3 × 1.2 cm. Abdominal ultrasonography revealed no evidence of hepatosplenomegaly.

**Figure 2:**
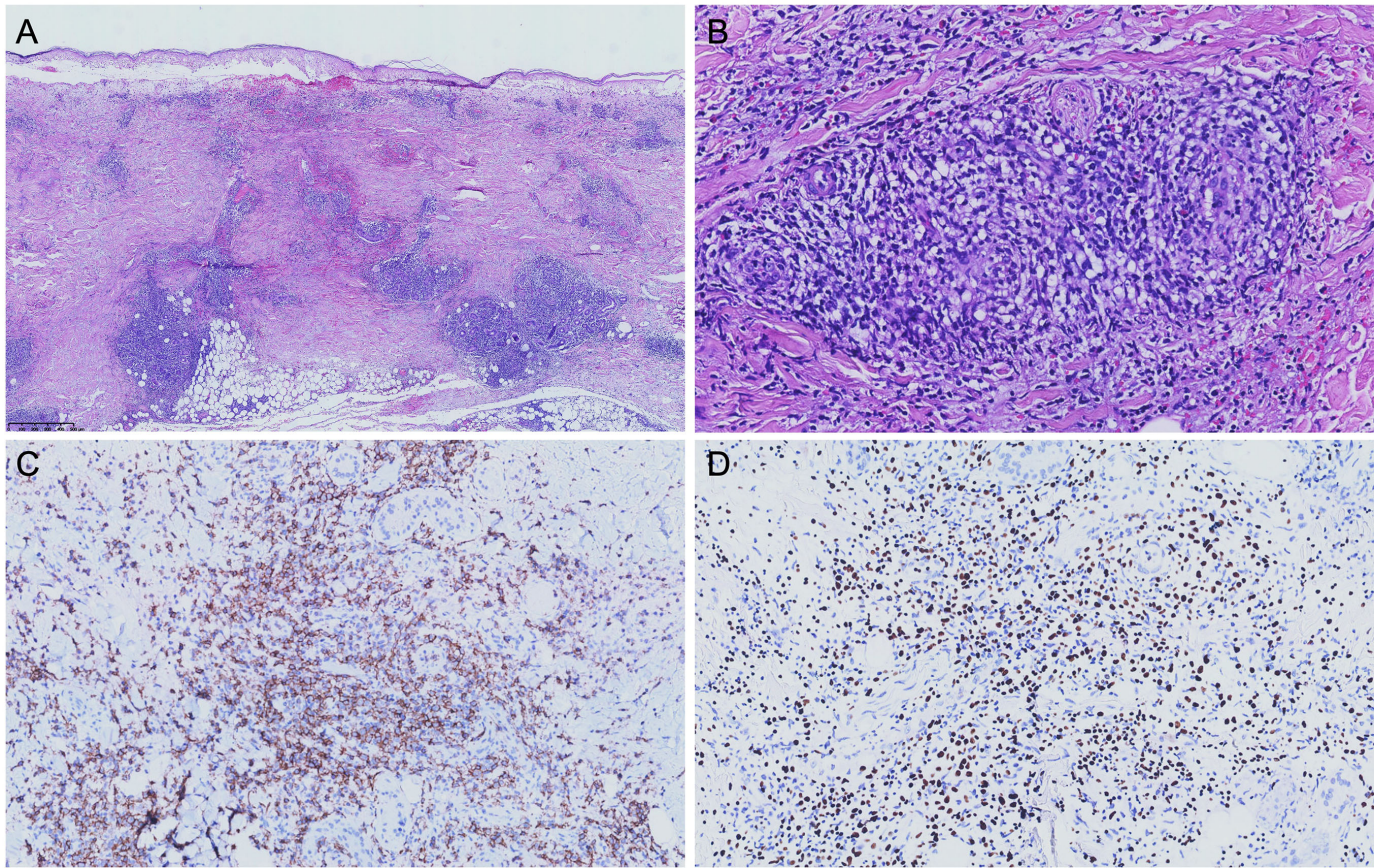
**(A, B)** Histopathologic examination of the skin biopsy specimen showed epidermal necrosis, interstitial, perivascular and periadnexal infiltration of lymphocytes, eosinophils and histiocytes with extension into the subcutaneous fat (a: H&E, 20×, b: 200×). **(C)** Immunohistochemical staining with CD56 (NK cell marker) was positive in skin lesion (200×). **(D)** *In situ* hybridization for EBER was positive (200×).

Skin biopsy displayed epidermal necrosis and a dense, diffuse infiltration of lymphocytes, eosinophils, and histiocytes. Notably, the lymphocytic infiltrate exhibited a prominent angiocentric pattern, intimately surrounding and permeating the vessel walls, accompanied by extension into the periadnexal structures and interlobular fat regions (**Figures 2A, B**). The lymphocytes stained positively for cytoplasmic CD3 (cCD3), CD7, CD56, Perforin, TIA1, LMP1, and Granzyme B, with scattered weak positivity for CD4, and were negative for CD8, CD20, CD79A, EBNA-2, PD1, and LEF1, indicating an NK phenotype (**Figure 2C**). The Ki-67 labeling index was 30%. *In situ* hybridization for EBV-encoded RNA (EBER) demonstrated diffuse positivity (**Figure 2D**). Further tests, including soluble CD25 levels, NK cell activity, and bone marrow biopsy, were recommended to definitively rule out HLH and hematologic malignancies but were declined by the patient and his family.

Based on the clinical features of SMBA, high EBV load, and histopathological findings, this patient was diagnosed with EBV-associated NK-cell LPD. Upon treatment with oral prednisone (30 mg/day), methotrexate (10mg/week), ganciclovir (600mg/day) and good ulcer care, the systemic symptoms resolved and the skin lesions improved substantially after 4 weeks. Given the risk of SMBA progressing to systemic disease, we strongly advised the patient to undergo regular monitoring during subsequent follow-ups. This routine surveillance included complete blood counts, blood biochemical tests, EBV DNA viral loads, serum ferritin, sCD25, coagulation profiles, and hepatosplenic and lymph node ultrasonography, with consideration for a bone marrow biopsy if clinically indicated. However, over the subsequent 2-year follow-up, the patient adhered only to regular evaluations of his skin rash, complete blood counts, and blood biochemical tests. Although he experienced recurrent cutaneous reactions following mosquito bites, no recurrence of systemic symptoms was observed, clinically supporting the absence of progressive HLH or overt systemic complications. A detailed timeline summarizing the patient’s clinical status, available quantitative laboratory parameters, and dynamic treatment adjustments throughout the 2-year follow-up is provided in **Supplementary Table 1**.

## Discussion

SMBA is currently classified under the spectrum of EBV-positive T/NK-cell lymphoproliferative disorders (EBV-T/NK-LPDs) and is recognized as a specific cutaneous manifestation of CAEBVD (2). The etiology of SMBA is intricately linked to the clonal proliferation of EBV-infected NK cells (2). EBV is a ubiquitous human herpesvirus with a lifelong latency in over 95% of the adult population (1). The pathogenesis of SMBA involves a complex interplay between mosquito salivary gland extracts (SGE) and the host immune system. Studies suggest that SGE stimulates the proliferation of SGE-specific CD4+ T cells, which subsequently induces the reactivation of latent EBV in NK cells and promotes their clonal expansion (10). As this clonal proliferation progresses, SMBA can evolve into life-threatening systemic conditions, such as systemic CAEBVD, hemophagocytic lymphohistiocytosis (HLH), or overt hematologic malignancies like extranodal NK/T-cell lymphoma (ENKTL) and aggressive NK-cell leukemia (3, 11–14).

Clinically, SMBA presents as edematous erythema, bullae, or hemorrhagic blisters at the bite sites, which rapidly progress to necrosis and ulceration, eventually healing with atrophic scars (2). Given the seasonal, bite-triggered pattern and a mixed eosinophilic infiltrate on skin biopsy, it is crucial to explicitly distinguish SMBA from the broader spectrum of exaggerated arthropod bite reactions or insect bite-like eruptions in hematologic patients, particularly eosinophilic dermatosis of hematologic malignancy (EDHM). EDHM is most commonly associated with B-cell malignancies, such as chronic lymphocytic leukemia. Clinically and histologically, EDHM can closely mimic arthropod reactions, presenting as seasonal, bite-triggered eruptions on exposed areas and characterized by superficial and deep perivascular and periadnexal eosinophil-rich infiltrates (15, 16). Unlike common arthropod bite reactions or EDHM, SMBA constitutes a distinct entity within EBV-positive T/NK-cell LPDs, driven directly by the proliferation of EBV-infected clonal cells. SMBA is distinguished by the precipitation of systemic manifestations, including high fever, general malaise, lymphadenopathy, hepatosplenomegaly, and liver dysfunction. Laboratory findings typically reveal elevated serum IgE levels, a high viral load of EBV DNA in peripheral blood, and NK-cell lymphocytosis. These fundamental differences underscore the necessity of evaluating EBV viral loads and NK/T-cell markers in such patients to ensure an accurate diagnosis and appropriate management.

Epidemiologically, SMBA is a rare condition with a strong predisposition for children and adolescents in East Asia; the mean age at diagnosis is approximately 6.7 ± 5.0 years (9). The prognosis is generally poor, with mortality primarily driven by HLH or progression to lymphoma. In this context, our case of a 70-year-old male represents a rare presentation of late-onset SMBA. To comprehensively review the clinical features of adult-onset SMBA, a literature search of the PubMed database was conducted from January 2000 to December 2025, using the search terms (“hypersensitivity to mosquito bites” OR “mosquito bite hypersensitivity” OR “severe mosquito bite allergy”) AND (“Epstein-Barr virus” OR “EBV”). The inclusion criteria were restricted to articles reporting EBV-associated SMBA cases in adult patients (aged >18 years) with sufficient clinical and laboratory data. Consequently, a total of 9 adult cases were identified and are summarized in **Table 1** (9, 11, 17–24).

**Table 1:** Summary of previously reported cases of SMBA in adults.

References	Age (years)	Skin clinical presentation	Systemic symptoms	Laboratory examination	Treatment	Complication (follow-up status)	Origin
Ohshima S et al. (17)	18	A 4-yr history of SMBA	Fever, malaise, hepatosplenomegaly	A marked increase of NK cell population, IgE 10,000 IU/mL, EBER (+)	NA	No signs of malignancy (NA)	NK
Cho JH et al. (18)	19	A history of SMBA since childhood	Fever	EBV anti-EA-DR IgG (+), anti-EBNA (+), EBV VCA IgG (+), NK cells increased (79%), many large granular lymphocytes in PBS	NA	NK-LGL lymphocytosis (NA)	NK
Tomita N et al. (20)	73	Ulceration at the site of mosquito bites in the lower thigh; soon she developed disseminated skin lesions characterized by redness, induration, and local heat; some lesions showed necrosis and ulceration, including those located in the nasal cavity	Fever and bilateral pretibial edema	Liver dysfunction, ferritin 10,318 ng/mL, EBER (+), CD56 (+). Southern analysis of the biopsy specimen showed an oligoclonal band representing EBV DNA.	Chemotherapy (DeVIC)	ENKTL, nasal type (died of lymphoma progression)	NK
Takeoka Y et al. (19)	31	A 28-yr history of SMBA	Fever, fatigue, hepatosplenomegaly	Pancytopenia, liver dysfunction, coagulopathy, ferritin 1969 ng/mL, IgE 9809 U/mL, EBV 6.0 × 10^5^ copies/mL	Immunochemotherapy, BMT	HLH (died of GVHD on the 92nd day after BMT)	NK
Chung JS et al. (21)	20	A history of SMBA since childhood	Fever, myalgia and lymphadenopathy	Many large granular lymphocytes in PBS, EBER (+), CD56 (+)	Chemotherapy (IMEP)	NK-LGL lymphoma (NA)	NK
Sugimoto KJ et al. (22)	52	A history of SMBA since childhood	Fever, nausea, anorexia, consciousness disorder, splenomegaly, and headaches	Liver dysfunction, thrombocytopenia, ferritin 666 ng/mL, EBV load 2.4 × 10^6^ copies/μgDNA (Peripheral mononuclear), EBV DNA 1.1 × 10^5^ copies/mL (plasma), EBER(+), CD56 (+), with LNs, liver, and spleen infiltration	Steroid	ANKL and cytotoxic T-cell lymphoma (died on the 8th day after admission)	T, NK
Kondo M et al. (24)	76	Granulomatous nodule gradually appeared on her right leg at the site of a possible mosquito bite after complete remission of ENKTL, Nasal Type	Negative	EBV DNA 240 copies/mL, EBER (+), CD56 (+)	Chemotherapy (GDP)	Localized NK/T-cell lymphoma (NA)	NK
Liu ZP et al. (23)	33	Swelling and blisters appeared around the left orbit after mosquito bites	Fever	High IgE level, EBV DNA 1500 copies/mL, EBER (+), CD56 (+)	Interferon	NA	T, NK
Higa M et al. (11)	20	A history of SMBA since childhood	Fever, lymphadenopathy, and splenomegaly	Multiple consolidations, mass-like lesions, nodules in both lungs, EBV DNA 3.466 Log IU/mL, EBER (+), CD56 (+)	Chemotherapy (SMILE), allo-HSCT	ENKTL (By day +186, the patient remained in remission)	NK

NK-LGL, NK cell-derived large granular lymphocyte; BMT, bone marrow transplantation; PBS, peripheral blood smear; ENKTL, extranodal natural killer/T-cell lymphoma; HLH, hemophagocytic lymphohistiocytosis; GVHD, graft versus host disease; SMILE chemotherapy, dexamethasone, methotrexate, ifosfamide, l-asparaginase, and etoposide; allo-HSCT, allogeneic hematopoietic stem cell transplantation; GDP chemotherapy, gemcitabine, cisplatin and dexamethasone; ANKL, aggressive natural killer leukemia; DeVIC, chemotherapy with carboplatin, etoposide, ifosfamide, and dexamethasone; IMEP, ifosfamide, methotrexate, etoposide, and prednisone.

Notably, in six of these cases, retrospective analysis revealed that SMBA had actually persisted since childhood. In the two confirmed cases of late-onset elderly patients, the disease followed an aggressive course, rapidly transforming into ENKTL (20, 24). The majority of adult patients (7/9) in our review developed severe complications such as HLH, ENKTL, or leukemia and succumbed to the disease during follow-up.

In contrast to these aggressive adult cases, our patient has exhibited a remarkably indolent clinical course. Over a 2-year follow-up period, despite recurrent localized necrotic reactions to mosquito bites, he has not experienced recurrent systemic symptoms such as fever or hepatosplenomegaly, nor has there been evidence of transformation into overt malignancy. This dissociation between the severe cutaneous phenotype and the indolent systemic progression highlights the heterogeneity of EBV-T/NK-LPDs. The prognosis of these disorders is highly variable; while some patients deteriorate rapidly, others maintain a stable condition for years without intervention. Recently, Akazawa et al. utilized multiomics analysis to identify a high-risk subtype of NK-cell type CAEBVD exhibiting tumor-like molecular characteristics, including a high CpG island methylation pattern(CIMP), a higher tumor mutational burden (TMB), and frequent copy number alterations (CNAs) (25). These findings underscore the heterogeneity of the disease at the genetic and epigenetic levels. Moving forward, the integration of next-generation sequencing technologies holds great promise for elucidating the molecular genetic landscape of CAEBVD. This approach could enable the prediction of distinct clinical courses and suggests possible molecular therapies.

Currently, there is no standardized treatment protocol for SMBA. The primary preventive measure is the avoidance of mosquito bites. Symptomatic relief can be achieved with corticosteroids, while immunomodulatory agents such as cyclosporine A, methotrexate, and etoposide are often employed to control the LPD (26). Antiviral agents like acyclovir and ganciclovir generally show limited efficacy because they target viral DNA polymerase, which is inactive in the latent phase of EBV infection. However, the therapeutic landscape is evolving. Novel immunotherapies, including PD-1 inhibitors (e.g., sintilimab), JAK inhibitors (e.g., ruxolitinib), and EBV-specific cytotoxic T lymphocytes (EBV-CTLs), have demonstrated promising efficacy and safety profiles in CAEBVD and related lymphomas. However, allogeneic hematopoietic stem cell transplantation (allo-HSCT) remains the only potentially curative therapy, a strategy supported by a recent breakthrough study by Wang et al., which identified EBV-infected hematopoietic stem cells (HSCs) as the cellular origin of CAEBVD (27, 28).

## Conclusion

We report a rare case of EBV-associated NK-cell LPD manifesting as SMBA in an elderly patient characterized by an unusually indolent course. Although our elderly patient exhibited no recurrence of systemic symptoms and no signs of malignant transformation during the 2-year follow-up, the risk of SMBA progressing to fatal hematologic malignancy remains a critical concern for such patients. Clinicians must maintain a high index of suspicion for underlying EBV-LPDs in elderly patients presenting with insect bite reactions. Long-term surveillance is mandatory to monitor for the potential evolution into aggressive lymphoma or HLH.

## Data Availability

The original contributions presented in the study are included in the article/**Supplementary Material**, further inquiries can be directed to the corresponding author.
